# Loss of Hepatic Surf4 Depletes Lipid Droplets in the Adrenal Cortex but Does Not Impair Adrenal Hormone Production

**DOI:** 10.3389/fcvm.2021.764024

**Published:** 2021-11-11

**Authors:** Xiaole Chang, Yongfang Zhao, Shucun Qin, Hao Wang, Bingxiang Wang, Lei Zhai, Boyan Liu, Hong-mei Gu, Da-wei Zhang

**Affiliations:** ^1^Institute of Atherosclerosis, College of Basic Medical Sciences, Shandong First Medical University, Shandong Academy of Medical Sciences, Tai'an, China; ^2^Department of Pediatrics and Group on the Molecular and Cell Biology of Lipids, Faculty of Medicine and Dentistry, University of Alberta, Edmonton, AB, Canada

**Keywords:** proprotein convertase subtilisin/kexin 9, LDL–cholesterol, cholesterol, triglyceride, atherosclerosis, LDL receptor (LDLR)

## Abstract

The adrenal gland produces steroid hormones to play essential roles in regulating various physiological processes. Our previous studies showed that knockout of hepatic Surf4 (Surf4^LKO^) markedly reduced fasting plasma total cholesterol levels in adult mice, including low-density lipoprotein and high-density lipoprotein cholesterol. Here, we found that plasma cholesterol levels were also dramatically reduced in 4-week-old young mice and non-fasted adult mice. Circulating lipoprotein cholesterol is an important source of the substrate for the production of adrenal steroid hormones. Therefore, we investigated whether adrenal steroid hormone production was affected in Surf4^LKO^ mice. We observed that lacking hepatic Surf4 essentially eliminated lipid droplets and significantly reduced cholesterol levels in the adrenal gland; however, plasma levels of aldosterone and corticosterone were comparable in Surf4^LKO^ and the control mice under basal and stress conditions. Further analysis revealed that mRNA levels of genes encoding enzymes important for hormone synthesis were not altered, whereas the expression of scavenger receptor class B type I (SR-BI), low-density lipoprotein receptor (LDLR) and 3-hydroxy-3-methyl-glutaryl-CoA reductase was significantly increased in the adrenal gland of Surf4^LKO^ mice, indicating increased *de novo* cholesterol biosynthesis and enhanced LDLR and SR-BI-mediated lipoprotein cholesterol uptake. We also observed that the nuclear form of SREBP2 was increased in the adrenal gland of *Surf4*^LKO^ mice. Taken together, these findings indicate that the very low levels of circulating lipoprotein cholesterol in Surf4^LKO^ mice cause a significant reduction in adrenal cholesterol levels but do not significantly affect adrenal steroid hormone production. Reduced adrenal cholesterol levels activate SREBP2 and thus increase the expression of genes involved in cholesterol biosynthesis, which increases *de novo* cholesterol synthesis to compensate for the loss of circulating lipoprotein-derived cholesterol in the adrenal gland of Surf4^LKO^ mice.

## Introduction

The adrenal cortex uses cholesterol as the substrate to produce steroid hormones, thus playing an indispensable role in regulating metabolism, water and salt balance and blood pressure, the immune system, stress response, and sexual development. Under a normal physiological condition, ~80% of cholesterol used in adrenal cortex hormone synthesis is derived from circulating lipoproteins, such as scavenger receptor class B, type I (SR-BI)-mediated selective uptake of cholesteryl ester from high-density lipoprotein (HDL) and low-density lipoprotein receptor (LDLR)-mediated endocytosis of LDL (1). The remaining cholesterol is contributed by *de novo* biosynthesis from acetate via the mevalonate pathway (2, 3), in which 3-hydroxy-3-methylglutaryl coenzyme A reductase (HMGCR) is the rate-limiting enzyme (4, 5). Cholesterol is stored as cholesteryl ester (CE) in lipid droplets in the adrenal cortex (2, 6, 7), which can be converted to free cholesterol by lipase-mediated lipolysis as needed for steroidogenesis (8–10). Free cholesterol from lipolysis of CE in lipid droplets, *de novo* biosynthesis, and plasma membrane can be rapidly transported to mitochondria by steroidogenic acute regulatory protein (StAR) (6, 8, 11, 12), where steroid hormones are synthesized from cholesterol by different mitochondrial P450 enzymes and then immediately released to circulation. Adrenal insufficiency characterized by low blood levels of cortisol and aldosterone can lead to a series of systemic clinical symptoms, including hypotension, anorexia, fatigue, syncope, hyponatremia, sexual dysfunction, mental disorders, etc. (3).

Surfeit 4 (Surf4) is a cargo receptor resided on the endoplasmic reticulum (ER) membrane, where it facilitates the transport of secretory proteins from the ER to the Golgi apparatus. Surf4 also mediates the retrograde transport of STING from the Golgi apparatus to the ER (13–19). We and others have found that Surf4 mediates secretion of very low-density lipoprotein (VLDL) (15, 19). Circulating VLDL is catabolized and eventually converted to LDL, which is then cleared from circulation mainly through hepatic LDLR (20). Knockout of LDLR (*Ldlr*^−/−^) in mice increases plasma cholesterol levels and risk for the development of atherosclerosis. Mutations in LDLR cause familial hypercholesterolemia (FH) in humans, which is characterized by elevated plasma LDL cholesterol levels and increased risk for cardiovascular disease. Current lipid-lowering drugs, such as statins and PCSK9 inhibitors, reduce plasma cholesterol levels mainly through increasing LDLR levels and thus LDL clearance. Therefore, they cannot effectively reduce plasma LDL cholesterol levels in FH patients.

Knockout of Surf4 in mouse liver (*Surf4*^LKO^) and knockdown of Surf4 in *Ldlr*^−/−^ mice significantly reduce VLDL secretion, leading to a drastic reduction in plasma cholesterol levels (15). However, we did not observe significant hepatic lipid accumulation or notable liver damage in *Surf4*^LKO^ mice or Surf4 knockdown *Ldlr*^−/−^ mice, indicating that hepatic Surf4 inhibition is a promising therapeutic target for lowering plasma lipids through suppressing LDL production. However, circulating lipoprotein-derived cholesterol, especially HDL cholesterol, is an important substrate for the production of adrenal cortex steroid hormones (1, 12, 21–24). Therefore, we investigated whether hepatic Surf4 silencing affected the production of adrenal cortex hormones. We found that lipid droplets and cholesterol levels were significantly reduced in the adrenal gland of *Surf4*^LKO^ mice compared to the control *Surf4*^Flox^ mice. However, plasma levels of adrenal cortex hormones, including corticosterone, aldosterone, and dehydroepiandrosterone (DHEA), and adrenocorticotropin (ACTH) were comparable in *Surf4*^LKO^ and *Surf4*^Flox^ mice. The expression of HMGCR, LDLR and SR-BI was markedly increased in the adrenal gland of *Surf4*^LKO^ mice. Therefore, knockout of hepatic Surf4 did not affect the production of adrenal cortex hormones despite a significant reduction in plasma and adrenal cholesterol levels.

## Materials and Methods

### Materials

H&E staining kit and saturated oil red O staining solution were purchased from Beijing Soleibao Technology Co., Ltd. (Beijing, China). Mouse corticosterone, aldosterone, DHEA, and ACTH ELISA kits were from Shanghai Enzyme Link Biotechnology Co., Ltd. (Shanghai, China). Total cholesterol kit was purchased from Qiyi Biotechnology Co., Ltd. (Shanghai, China). Anti-LDLR, HMGCR, SR-BI, and SREBP-2 antibodies were from Abcam. Anti-β-actin and ACAT1 antibodies were from Beijing Boaosen Biotechnology Co., Ltd. (Beijing, China) and Proteintech, respectively. Horseradish enzyme-labeled goat anti-rabbit or mouse IgG was purchased from Beijing Zhongshan Jinqiao Biotechnology Co., Ltd. (Beijing, China). RNAprep Pure Tissue Kit, EasyQuick RT MasterMix, and Top Green qPCR SuperMix were purchased from Tiangen Biochemical Technology Co., Ltd. (Beijing, China), Kangwei Century Biotechnology Co., Ltd. (Beijing, China), and Beijing Quanshijin Biotechnology Co., Ltd. (Beijing, China), respectively.

### Animal

*Sur4*^Flox^ and *Surf4*^LKO^ mice in C57BL/6 background were generated as described (15) and were maintained in the animal facility at Shandong First Medical University (Taian, China). Three to five mice were housed per cage with free access to H_2_O in a climate-controlled facility with a 12-h light/dark cycle. After weaning, mice were fed *ad libitum* a chow diet containing 20% protein, 5% fat, and 48.7% carbohydrates (Keao Xieli, Beijing, China). All animal procedures were approved by Shandong First Medical University's Animal Care and Use Committee.

### Histochemistry

The experiments were performed as described (15, 25). Briefly, for H&E staining, tissues were fixed, embedded in paraffin, cut into 8 μm, and mounted on slides. After, the sections were deparaffinized, rehydrated, and then stained with hematoxylin and eosin sequentially. For Oil Red-O staining, fresh tissue samples were embedded in Optimal Cutting Temperature compound, cut into 10 μm, and then mounted on slides. After, the sections were fixed in formalin, stained with Oil Red-O and then hematoxylin. All slices were imaged on a microscope (Nikon, Tokyo, Japan). Relative stained areas were quantified with ImageJ software (National Institute of Health) using color segmentation and threshold analysis.

### ELISA

Blood samples were collected from fasted or non-fasted mice. Plasma levels of adrenal hormones and ACTH were measured with their specific ELISA kits according to the manufacturer's instruction. The optical density was measured using a SpectraMax i3x Microplate Reader (Filter: 450 nm).

### Quantitative Real-Time PCR

Total RNAs were extracted from mouse tissues using RNAprep Pure Tissue Kit according to the manufacturer's protocols. Complementary DNA (cDNA) was synthesized using EasyQuick RT MasterMix from Kangwei Century Company (China). qRT-PCR was carried out using Top Green qPCR SuperMix. 2^−ΔΔct^ was used to analyze relative gene expression. *Gapdh* was the control. The primers were designed and synthesized by Shanghai Shenggong Biological Engineering Co., Ltd. and listed in Table 1.

**Table 1:** Sequences of the primers.

**Name**	**Primer sequence**	**Product (bp)**
StAR	Forward: 5′-TTGGGCATACTCAACAACCAG-3′	195
	Reverse: 5′-GACATTTGGGTTCCACTCTCC-3′	
*Cyp11a1*	Forward: 5′-GGTGTAGCTCAGGACTTCATCAAA-3′	109
	Reverse: 5′-ACTCAAAGGAAAAGCGGAATAGG-3′	
*Cyp21a2*	Forward: 5′-CTCCGGCTATGACATCCCTA-3′	151
	Reverse: 5′-ACAGCCAAAGGATGGTGTTC-3′	
*Cyp11b1*	Forward: 5′-GTATCGAGAGCTGGCAGAGG-3′	140
	Reverse: 5′-GGGTTGATGTCGTGTCAGTG-3′	
*Cyp11b2*	Forward: 5′-CTGAACGCTATATGCCTCAGC-3′	160
	Reverse: 5′-AGTGTCTCCACCTGGAAGGTT-3′	
*Ldlr*	Forward: 5′-ACCCGCCAAGATCAAGAAAG-3′	148
	Reverse: 5′-GCTGGAGATAGAGTGGAGTTTG-3′	
*Hmgcr*	Forward: 5′-GCCCTCAGTTCAAATTCACAG-3′	96
	Reverse: 5′-TTCCACAAGAGCGTCAAGAG-3′	
*Scarb1*	Forward: 5′-CCCTATTCCATTGACTCTGAGC-3′	121
	Reverse: 5′-CACATAAGAGGATTCGAGAGCG-3′	
*Hmgcs1*	Forward: 5′-GCGTCTTTGCTTGTGTCTAATC-3′	125
	Reverse: 5′-GAGAACACTCCAACCCTCTTC-3′	
*Mvk*	Forward: 5′-GGAGCAACTGGAGAAGCTAAA-3′	100
	Reverse: 5′-TGCCAGGTACAGGTAGAGAA-3′	
*Fdps*	Forward: 5′-TCGGGTGAAAGCACTGTATG-3′	100
	Reverse: 5′-GCACTGCTCTATGAGACTCTTG-3′	
*Fdft1*	Forward: 5′-CTCACCTGAAAGCCCAGAAA-3′	96
	Reverse: 5′-CCTGCTTTCCTTACCCTCATC-3′	
*Gapdh*	Forward: 5′-AACTTTGGCATTGTGGAAGG-3′	132
	Reverse: 5′-GGATGCAGGGATGATGTTCT-3′	

### Immunoblotting

Tissue samples were collected from euthanized mice and homogenized in RIPA buffer (50 mM Tris, pH 7.4, 150 mM NaCl, 1% TritonX-100, 1% sodium deoxycholate, 0.1% SDS, sodium orthovanadate, sodium fluoride, EDTA, leupeptin, and PMSF) as described (15). The supernatant was harvested as tissue homogenate after centrifugation. Protein concentrations were determined by the BCA protein assay. Equal amounts of total lysate proteins were applied to SDS-PAGE and then transferred to PVDF membranes (Millipore) by electroblotting. Immunoblotting was performed using specific antibodies as indicated. Antibody binding was detected by HRP-conjugated goat anti-mouse or rabbit IgG antibody, followed with Pierce™ ECL Western Blotting Substrate. The image was acquired and analyzed on a Tanon 5200 automatic chemiluminescence image analysis system. The densitometry was quantified in Image-Pro Plus.

### Plasma and Tissue Lipids

Blood samples were collected into EDTA-coated tubes from mice and then centrifuged at 3000 x g for 10 min. Plasma from each mouse was subjected to analysis of triglyceride (TG), total cholesterol (TC), and HDL-C using their specific kits according to the manufacturer's instructions (Applygen, Beijing, China).

Adrenal lipids were extracted using the methyl-tert-butyl ether (MTBE) method as described (15). Briefly, tissues (3–5 mg) were homogenized in 280 μl of cold methanol containing an internal standard of cholesteryl ester (CE) 17:0. In total, 50 μl of homogenates were stored to analyze protein content using a commercial BCA protein assay kit (Solarbio Co., Beijing, China). MTBE (1 ml) was added to the remaining volume of homogenates; the samples were vortexed, rotated for 1 h at room temperature, and then mixed with 325 μl H_2_O. After, the samples were centrifuged at 10,000 × g for 10 min at 4°C. The upper hydrophobic fraction was collected and dried under nitrogen gas. The samples were dissolved in methanol. Total cholesterol and free cholesterol were measured using an enzymatic kit from Biosino Biotechnology (Beijing, China) and Applygen (Beijing, China), respectively.

Dried lipid extracts were also reconstituted in 200 μl of acetonitrile:2-propanol (1:1, v/v) and then subjected to liquid chromatography tandem mass spectrometry (LC-MS/MS) analysis as described (15). Briefly, LC-MS/MS was performed using a Shimadzu LC-20 AD binary pump system coupled to a SIL-20AC autoinjector and interfaced with an ABI 4000 QTrap mass spectrometer (Sciex, Framingham, MA, USA). Chromatographic separations were carried out on a Waters Symmetry C18 column with a Waters C18 guard column. The mobile phase comprised (A) 10 mM ammonium formate in acetonitrile: water: formic acid (83:17:0.1, v/v/v) and (B) 10 mM ammonium formate in acetonitrile: 2-propanol: formic acid (50:50:0.1, v/v/v). Isocratic elution was performed with 95% B for 16 min. Relative quantification of lipids in samples was carried out based on the intensity of each species divided by the intensity of the internal standards and protein concentrations.

### Statistical Analysis

All statistical analyses were carried out by GraphPad Prism version 9.0 (GraphPad Software). The significant differences between groups were determined via a Student's *t*-test or a Mann Whitney test. All data met normal distribution criteria and variance between groups that was analyzed by F-test showed no significant difference (*P* > 0.05). Values of all data, unless otherwise indicated, were depicted as mean ± S.D. All experiments, unless indicated, were repeated at least three times. The significance was defined as ^*^p < 0.05, ^**^p < 0.01, ^***^p < 0.001, and ^****^p < 0.0001.

## Results

### Effect of Hepatic Surf4 Silencing on Cholesterol Levels in the Adrenal Gland

**Figure 1:**
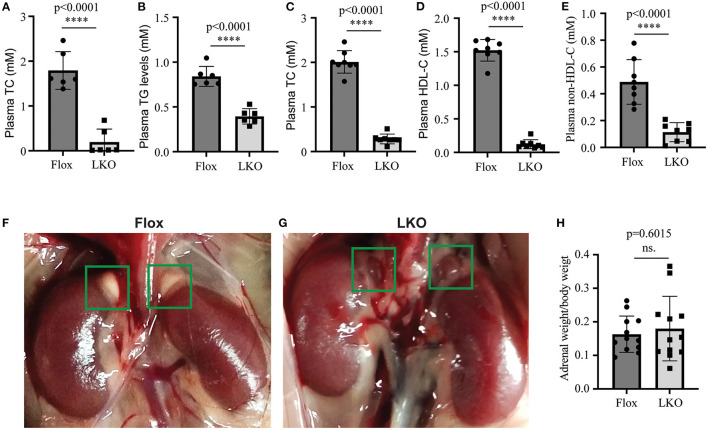
The effect of hepatic knockout of Surf4 on plasma lipids and the adrenal gland. **(A–E)** Plasma lipid levels. Blood samples were collected from 4-week-old mice **(A,B)** fasted for 10 h or 14-week-old mice fasted for 10 h and refed for 4 h **(C–E)**. Plasma levels of total cholesterol (TC), triglycerides (TG), and HDL cholesterol (HDL-C) were measured using their specific enzymatic kits (*n* ≥ 6). Non-HDL-C was calculated by subtracting HDL-C from TC. **(F,G)** Representative images of the adrenal glands of *Surf4*^Flox^ **(F)** and *Surf4*^LKO^ mice **(G)** (green frame). Similar results were observed in other mice. **(H)** Ratio of the adrenal weight to the body weight of the same mouse. Values of all data were mean ± S.D. The significance was defined as ****p < 0.0001 and *P* > 0.05, no significance (ns.).

We have reported that knockout of hepatic Surf4 impairs VLDL secretion and markedly reduces fasting plasma cholesterol and TG levels in adult mice (15). Here, we found that fasting plasma total cholesterol and TG levels were also dramatically reduced in 4-week-old young *Surf4*^LKO^ mice (Figures 1A,B). Furthermore, similar to the results of the study on fasting plasma cholesterol levels (15), non-fasting plasma levels of total cholesterol, HDL cholesterol and non-HDL cholesterol were all markedly reduced in *Surf4*^LKO^ mice (Figures 1C–E). Therefore, the lack of hepatic Surf4 drastically reduced plasma cholesterol levels in young mice and fasted or non-fasted adult mice. Considering that circulating lipoprotein cholesterol is an important substrate for adrenal steroid hormone biosynthesis, we assessed whether the adrenal gland was affected in *Surf4*^LKO^ mice. As shown in Figures 1F,G, the adrenal glands in *Surf4*^Flox^ and *Surf4*^LKO^ mice were similar in size and shape. The ratio of adrenal weight to body weight was also comparable in the two genotypes (Figure 1H). However, their colors were different, white and red in *Surf4*^Flox^ and *Surf4*^LKO^ mice, respectively.

**Figure 2:**
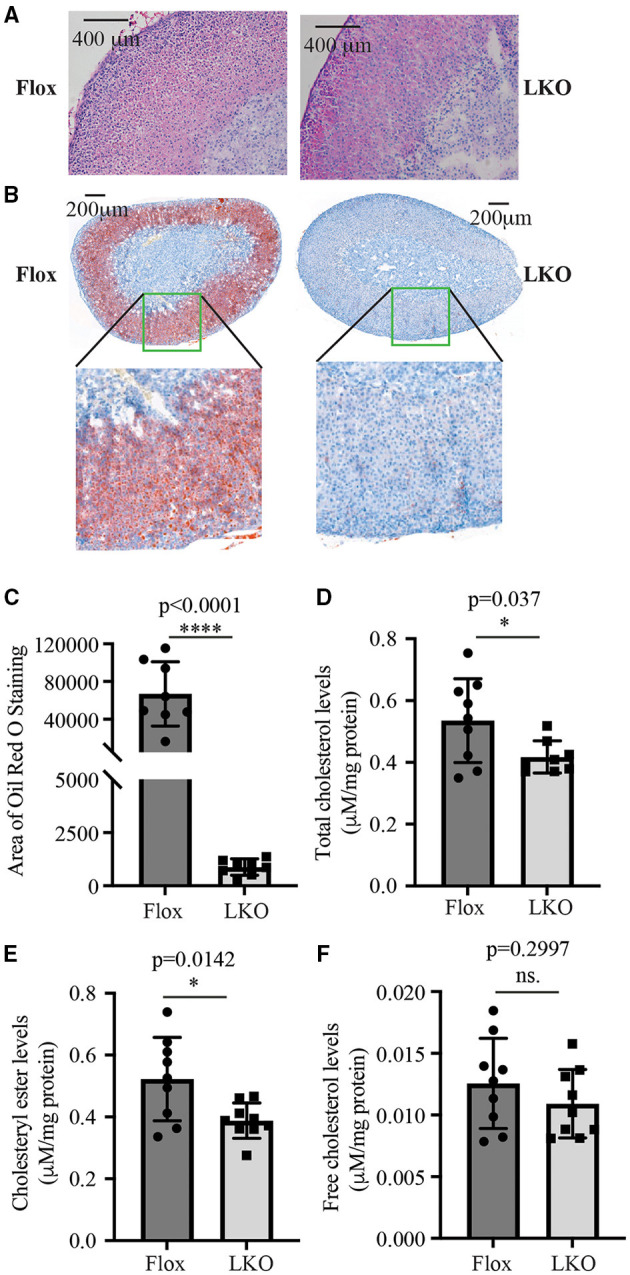
Impact of lacking hepatic Surf4 on the adrenal gland. **(A)** H&E staining of adrenal sections. **(B,C)** Oil Red O of adrenal sections. Representative figures were shown. Similar results were obtained in other mice. The images of Oil Red O staining were quantified using ImageJ **(C)** (8 mice per group). Data were analyzed by a Student's *t*-test (*p* < 0.0001) and a Mann Whitney test (*p* = 0.0002). **(D–F)** Adrenal total cholesterol **(D)**, cholesteryl ester **(E)**, and free cholesterol **(F)** levels. Lipids were extracted from mouse adrenal glands using the MTBE method and then subjected to total cholesterol and free cholesterol measurement using their specific enzymatic kit. Cholesteryl ester levels were calculated by subtracting free cholesterol from total cholesterol. Values of all data were mean ± S.D. The significance was defined as *p < 0.05 and ****p < 0.0001, *p* > 0.05, no significance (ns.).

Next, we used H&E and Oil Red O staining to analyze the histology of the adrenal gland. As shown in Figure 2A, the adrenal cortex of *Surf4*^Flox^ mice displayed many vacuoles, which were essentially absent in the adrenal cortex of *Surf4*^LKO^ mice. Oil Red O staining revealed that the adrenal cortex of *Surf4*^Flox^ mice contained many lipid droplets stained red (Figure 2B). Conversely, in the adrenal gland of *Surf4*^LKO^ mice, the staining appeared as small puncta and was markedly reduced (Figures 2B,C), and the levels of total cholesterol and cholesteryl ester (CE) were also significantly reduced (Figures 2D,E). However, adrenal free cholesterol levels were not significantly affected by hepatic knockout of Surf4 (Figure 2F). A detailed analysis of adrenal lipids using LC-MS/MS revealed that the levels of CE18:1, CE20:4, and CE22:6, but not CE 16:1, CE18:0, CE18:2, CE18:3, CE20:3, or CE20:5 were significantly reduced in the adrenal gland of *Surf4*^LKO^ mice (Table 2). Therefore, lacking hepatic Surf4 significantly reduced cholesteryl ester levels and virtually depleted lipid droplets in the adrenal gland, which could explain the color change observed in the adrenal gland of *Surf4*^LKO^ mice.

**Table 2:** Adrenal cholesteryl ester levels.

**CE**	**Flox**	**LKO**	***P*-value**
CE16:1	0.036 ± 0.013	0.032 ± 0.018	0.6957
CE18:0	0.306 ± 0.293	0.261 ± 0.214	0.7737
**CE18:1**	**0.402** **±** **0.111**	**0.181** **±** **0.159**	**0.0191**
CE18:2	0.84 ± 0.323	0.49 ± 0.379	0.1153
CE18:3	0.88 ± 0.864	0.578 ± 0.597	0.4085
CE20:3	6.046 ± 2.961	3.011 ± 3.75	0.1091
**CE20:4**	**9.225** **±** **5.653**	**2.33** **±** **2.914**	**0.0141**
CE20:5	4.764 ± 5.591	2.257 ± 3.939	0.3321
**CE22:6**	**31.272** **±** **24.202**	**7.56** **±** **8.549**	**0.0219**

*Lipids were extracted from the adrenal gland using MTBE and then analyzed with LC-MS/MS. Relative quantification of lipids in samples was carried out based on the intensity of each species divided by the intensity of the internal standard (CE17:0) and protein concentrations. Values of all data were mean ± S.D. The significance was defined as p < 0.05 (shown in bold)*.

### Effect of Hepatic Surf4 Knockout on the Production of Adrenal Cortex Hormones

**Figure 3:**
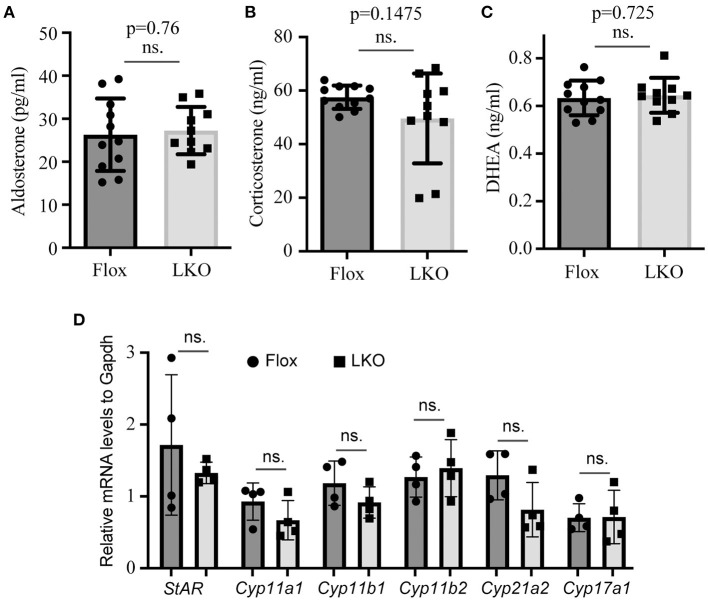
Impact of lacking hepatic Surf4 on plasma levels of adrenal hormones. **(A–C)** Plasma adrenal hormone levels. Blood samples were collected from non-fasted mice [*Surf4*^Flox^ (Flox) and *Surf4*^LKO^ (LKO) mice, 10–14-week-old]. Aldosterone **(A)**, corticosterone **(B)**, and DHEA **(C)** were measured using their specific ELISA kits. **(D)** qRT-PCR. Total RNAs were extracted from adrenal glands and then subjected to qRT-PCR. The relative mRNA levels were the ratio of the target's mRNA levels indicated to that of *Gapdh* at the same condition (*n* = 4). Values of all data were mean ± S.D. *p* > 0.05 was defined as no significant difference (ns.).

The adrenal cortex uses cholesterol as the substrate to produce steroid hormones. Therefore, we used ELISA to measure the plasma levels of aldosterone, corticosterone and DHEA, and found that their levels were comparable in non-fasted *Surf4*^Flox^ and *Surf4*^LKO^ mice (Figures 3A–C). We then used qRT-PCR to assess the expression of genes that are crucial for adrenal cortex hormone synthesis. StAR mediates the transport of cholesterol from the outer to the inner membrane of the mitochondrial and is believed to be the rate-limiting step in adrenal corticosteroid synthesis. Cyp11a1 converts cholesterol to pregnenolone. Cyp17a1 is involved in the last step of DHEA synthesis. Cyp11b1 and Cyp11b2 are the key enzymes for aldosterone synthesis, and Cyp21a2 and Cyp11b1 are crucial for corticosteroid synthesis. qRT-PCR data showed that the mRNA levels of these enzymes were not significantly altered in the adrenal gland of *Surf4*^LKO^ mice (Figure 3D). Taken together, these findings indicate that under basal conditions, the production of adrenal cortex hormone in *Surf4*^LKO^ mice is not significantly affected.

**Figure 4:**
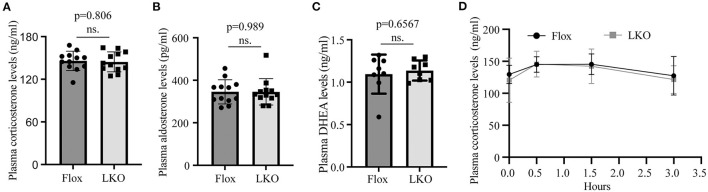
Plasma levels of adrenal steroid hormones. **(A–C)** Fasting plasma hormone levels. Mice were fasted for 12 h, and blood samples were collected for the measurement of corticosterone **(A)**, aldosterone **(B)**, and DHEA **(C)** with their specific ELISA kits. **(D)** Plasma corticosterone levels. Mice fasted for 12 h were subjected to cold stimulation (4°C). Blood samples were collected at the indicated time points. Plasma corticosterone levels were measured using its specific ELISA kit (*n* = 6). Values of all data were mean ± S.D. *p* > 0.05 was defined as no significant difference (ns.).

Stress stimuli, such as fasting and cold exposure, can stimulate the pituitary to release ACTH, which then activates the adrenal cortex to produce corticosterone (12, 26, 27). To evaluate whether the production of adrenal steroid hormones was affected in *Surf4*^LKO^ mice under stress conditions, we subjected mice to fasting and found that the levels of fasting plasma corticosterone, aldosterone and DEHA were comparable in *Surf4*^Flox^ and *Surf4*^LKO^ mice (Figures 4A–C). We then challenged fasted mice with acute cold stimulation at 4°C for up to 4 h. As shown in Figure 4D, the plasma levels of corticosterone were comparable and displayed a similar pattern in *Surf4*^Flox^ and *Surf4*^LKO^ mice, increasing after cold stimulation until the 0.5-h time point and then gradually decreasing at the 1.5-h time point. Therefore, the production of corticosterone under stress stimuli is not affected by hepatic Surf4 knockout.

### Effect of Hepatic Surf4 Knockout on the Expression of Genes Involved in Cholesterol Metabolism

**Figure 5:**
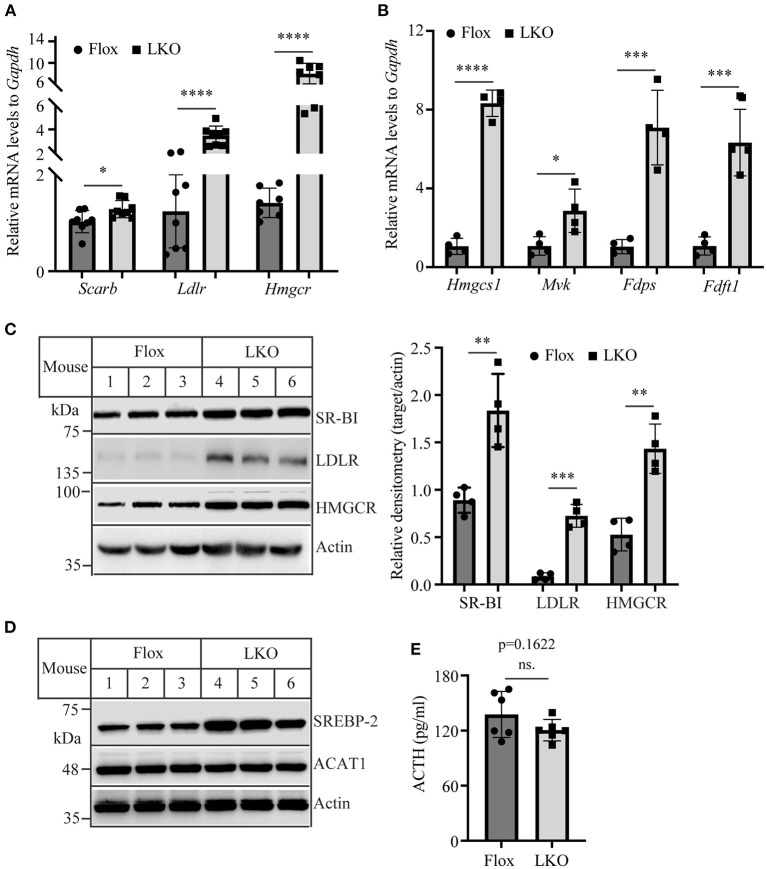
The expression of genes involved in cholesterol metabolism. **(A,B)** qRT-PCR. The relative mRNA levels were the ratio of the target's mRNA levels indicated to that of *Gapdh* at the same condition. **(C,D)** Immunoblotting. The same amount of adrenal homogenate was applied to Western Blot using antibodies as indicated. Representative figures were shown. The densitometry was assessed. The relative densitometry is the densitometry of each target to that of actin in the same sample **(C)**. **(E)** Plasma levels of ACTH. Mice (10–12-week-old, male) were fasted for 12 h. Plasma was then collected and subjected to the measurement of ACTH using an ELISA kit. Values of all data were mean ± S.D. The significance was defined as *p < 0.05, **p < 0.01, ***p < 0.001, and ****p < 0.0001. *p* > 0.05 was defined as no significant difference (ns.).

Adrenal cells take up circulating LDL and HDL cholesterol via LDLR and SR-BI, respectively. They also produce cholesterol via *de novo* biosynthesis. Therefore, we examined the expression of LDLR, SR-BI, and HMG-CoA reductase, the latter being the rate-limiting enzyme in cholesterol biosynthesis. qRT-PCR showed that the mRNA levels of *Scarb, Ldlr*, and *Hmgcr* were all significantly increased in the adrenal gland of *Surf4*^LKO^ mice (Figure 5A). We also measured the mRNA levels of additional genes involved in cholesterol biosynthesis, including HMG-CoA synthase (*Hmgcs1*), mevalonate kinase (*Mvk*), farnesyl diphosphate synthase (*Fdps*), and squalene synthase (*Fdft1*), and observed that they were all significantly increased in the adrenal gland of *Surf4*^LKO^ mice (Figure 5B). Consistently, western blot data revealed an increase in the protein levels of adrenal SR-BI, LDLR and HMGCR in *Surf4*^LKO^ mice (Figure 5C). SREBP2 upregulates the transcription of genes involved in cholesterol metabolism, such as LDLR, HMGCR, HMG-CoA synthase, squalene synthase, etc. (28). Acyl-CoA:cholesterol acyltransferase 1 (ACAT1) converts free cholesterol to cholesteryl ester. Lacking ACAT1 significantly reduces cellular cholesteryl ester levels (29). Therefore, we measured adrenal SREBP2 and ACAT1 levels. As shown in Figure 5D, the levels of the nuclear form of SREBP2 were increased, while ACAT1 levels were not changed in the adrenal gland of *Surf4*^LKO^ mice compared to *Surf4*^Flox^ mice. In addition, ACTH can increase expression of LDLR and SR-BI (30, 31). Therefore, we assessed plasma ACTH levels in *Surf4*^Flox^ and *Surf4*^LKO^ mice using ELISA. As shown in Figure 5E, lacking hepatic Surf4 did not significantly affect plasma ACTH levels. Taken together, these findings suggest an increased ability in cholesterol *de novo* biosynthesis and uptake of circulating lipoproteins in the adrenal gland of *Surf4*^LKO^ mice.

## Discussion

Here, we found that (1) knockout of hepatic Surf4 in mice essentially depleted lipid droplets and significantly reduced cholesteryl ester levels in the adrenal glands of *Surf4*^LKO^ mice; (2) the production of adrenal cortex steroid hormones was comparable in *Surf4*^LKO^ and *Surf4*^Flox^ mice under basal and stress conditions; and (3) the expression of genes involved in steroid hormone synthesis was not affected, whereas the expression of SREBP2 and genes involved in cholesterol metabolism was significantly increased in the adrenal gland of *Surf4*^LKO^. Taken together, these findings indicate that the ability of the adrenal cortex to produce steroid hormones is not affected by the lack of hepatic Surf4 even though plasma lipoprotein cholesterol levels are drastically reduced in the young and fasted and non-fasted adult mice.

VLDL is exclusively secreted by hepatocytes and can be catabolized into LDL in circulation. Hepatic knockout of Surf4 virtually eliminates VLDL secretion and LDL production in mice. LDLR-mediated LDL endocytosis can provide cholesterol for adrenal steroidogenesis (24, 32). However, several lines of evidence show that SR-BI-mediated selective CE uptake from HDL serves as an important source of cholesterol for adrenal cortex steroidogenesis under stress conditions (1, 12, 21–23). In addition, Bochem et al. reported that HDL-derived cholesterol is an important substrate for adrenal cortex hormone synthesis under a basal condition in humans (33). Knockout of apolipoprotein A-I (apoA-I), the main structural lipoprotein on HDL, reduced plasma HDL-C levels by ~74% and essentially depleted lipid droplets in the adrenal cortex. The production of corticosteroids under a basal or stress condition was also significantly reduced in apoA-I knockout mice (34–36). Like apoA-I knockout mice, *Surf4*^LKO^ mice display a similar reduction in plasma HDL cholesterol levels under fasting and non-fasting conditions. However, the production of adrenal cortex hormones in *Surf4*^LKO^ mice was not significantly affected even under stress conditions.

SR-BI mediates the selective uptake of CE from HDL (23, 37). Knockout of SR-BI significantly reduces the production of glucocorticoids under stress conditions even though plasma levels of apoA-I and HDL cholesterol levels remain unchanged and are increased, respectively (1, 21, 37). SR-BI can bind to HDL particles reconstituted with apoA-I, apoA-II, apoE, and apoC-III (38); however, lacking apoA-I significantly reduces the capacity of SR-BI to mediate CE-selective uptake from HDL. Furthermore, the accumulation of CE in steroidogenic cells is essentially disappeared in apoA-I knockout mice (34, 35, 39). These findings indicate that apo A-I is essential for the effective transfer of CE from HDL to adrenal cortex cells. *Surf4*^LKO^ mice display a significant reduction in plasma levels of HDL cholesterol and apoA-I; however, unlike apoA-I knockout mice that are completely deficient in apoA-I, *Surf4*^LKO^ mice still retain a small portion of circulating apoA-I and HDL cholesterol (15). The increased expression of SR-BI in the adrenal gland of *Surf4*^LKO^ mice may enhance the ability of adrenal cortex cells to take up cholesterol from residual circulating HDL, thereby mitigating the adverse effect of decreased plasma cholesterol levels on adrenal cortex steroidogenesis.

Cholesterol biosynthesis provides ~20% of the substrate for steroidogenesis in the adrenal cortex under normal conditions. Total cholesterol levels in the adrenal gland of *Surf4*^LKO^ mice were significantly reduced, leading to increased levels of the nuclear form of SREBP2. This upregulated the expression of genes involved in cholesterol *de novo* biosynthesis and LDLR. LDLR mediates endocytosis of LDL and apoE-containing lipoprotein particles, such as VLDL and chylomicron remnants. Plasma apoE levels are comparable in *Surf4*^LKO^ and *Surf4*^Flox^ mice (15). Together, our findings suggest that cholesterol biosynthesis and LDLR-mediated endocytosis of apoE-containing lipoproteins may increase in adrenal cortex cells of *Surf4*^LKO^ mice, which at least partially compensate for the loss of HDL-derived cholesterol.

Deficiency of hepatic Surf4 significantly reduces VLDL secretion, plasma cholesterol levels and the development of atherosclerosis but does not cause notable liver damage or hepatic lipid accumulation in mice (15). Here, we found that knockout of hepatic Surf4 drastically reduces plasma cholesterol in 4-week-old young mice (newly weaned) and non-fasted adult mice, whereas the production of adrenal steroid hormones is not significantly impaired in *Surf4*^LKO^ mice under normal and stress conditions. These findings indicate that hepatic Surf4 inhibition is a promising therapeutic target for lowering plasma lipid levels in patients whose disease cannot be effectively managed by currently available strategies.

## Data Availability Statement

The original contributions presented in the study are included in the article/supplementary material, further inquiries can be directed to the corresponding author/s.

## Ethics Statement

The animal study was reviewed and approved by Shandong First Medical University's Animal Care and Use Committee.

## Author Contributions

SQ and D-wZ designed the experiments, analyzed data, and wrote the manuscript. XC and ZY performed the experiments, analyzed data, and wrote the first draft. HW, BW, LZ, YZ, BL, and H-mG performed experiments and analyzed data. All authors contributed to the article and approved the submitted version.

## Funding

This work was supported by National Natural Science Foundation of China (NSFC 81929002), Academic Promotion Program of Shandong First Medical University (2019QL010 and 2019PT009), and The Natural Sciences and Engineering Research Council of Canada (RGPIN-2016-06479). D-wZ was also supported by grants from Canadian Institutes of Health Research (PS 178091) and the China Institute at the University of Alberta. SQ was supported by 91539114 and ts201511057.

## Conflict of Interest

The authors declare that the research was conducted in the absence of any commercial or financial relationships that could be construed as a potential conflict of interest.

## Publisher's Note

All claims expressed in this article are solely those of the authors and do not necessarily represent those of their affiliated organizations, or those of the publisher, the editors and the reviewers. Any product that may be evaluated in this article, or claim that may be made by its manufacturer, is not guaranteed or endorsed by the publisher.
