# Corrigendum: Loss of hepatic Surf4 depletes lipid droplets in the adrenal cortex but does not impair adrenal hormone production

**DOI:** 10.3389/fcvm.2023.1215335

**Published:** 2023-05-10

**Authors:** Xiaole Chang, Yongfang Zhao, Shucun Qin, Hao Wang, Bingxiang Wang, Lei Zhai, Boyan Liu, Hong-mei Gu, Da-wei Zhang

**Affiliations:** ^1^ Institute of Atherosclerosis, College of Basic Medical Sciences, Shandong First Medical University, Shandong Academy of Medical Sciences, Tai’an, China; ^2^ Department of Pediatrics and Group on the Molecular and Cell Biology of Lipids, Faculty of Medicine and Dentistry, University of Alberta, Edmonton, AB, Canada

**Keywords:** proprotein convertase subtilisin/kexin 9, LDL-cholesterol, cholesterol, triglyceride, atherosclerosis, LDL receptor (LDLR)

A Corrigendum on Loss of hepatic Surf4 depletes lipid droplets in the adrenal cortex but does not impair adrenal hormone production By Chang X, Zhao Y, Qin S, Wang H, Wang B, Zhai L, Liu B, Gu H-m and Zhang D-w. (2021) Front. Cardiovasc. Med. 8:764024. doi: 10.3389/fcvm.2021.764024

In the published article, there was an error in the Funding statement. Instead of “the China Institute at the University of China” it should be “the China Institute at the University of Alberta.” The correct Funding statement appears below.

## FUNDING

This work was supported by National Natural Science Foundation of China (NSFC 81929002), Academic Promotion Program of Shandong First Medical University (2019QL010 and 2019PT009), and The Natural Sciences and Engineering Research Council of Canada (RGPIN-2016-06479). D-wZ was also supported by grants from Canadian Institutes of Health Research (PS 178091) and the China Institute at the University of Alberta. SQ was supported by 91539114 and ts201511057.

The authors apologize for this error and state that this does not change the scientific conclusions of the article in any way. The original article has been updated.

